# Patient characteristics, disease manifestations and diagnostic findings of consecutive patients suspected of Giant Cell Arteritis (GCA) — retrospective experience from a fast-track clinic in Israel

**DOI:** 10.1007/s10067-025-07787-0

**Published:** 2025-11-27

**Authors:** Amir Haddad, Tal Gazitt, Ameen Batheesh, Joy Feld, Naomi Yeshurun Finkelstein, Muna Elias, Muhanad Abu Elhija, Noa Hayat, Devy Zisman

**Affiliations:** 1 Technion-Israel Institute of Technology, Rappaport Faculty of Medicine, Haifa, Israel; 2 Rheumatology Unit, Carmel Medical Center, 7 Michal Street, Haifa, Israel

**Keywords:** Fast-track clinic (FTC), GCA mimickers, Giant cell arteritis (GCA), Probability score, Ultrasound

## Abstract

**Introduction/objective:**

Giant Cell Arteritis (GCA) is relatively rare in the Middle East compared to Western countries. We aimed to retrospectively investigate the demographic, clinical, and imaging characteristics and the definitive diagnoses of patients referred to a GCA fast-track clinic (FTC) in the northwest of Israel with a mixed population of Arabs and Jews.

**Methods:**

Demographic, clinical and laboratory data of patients referred to GCA-FTC between 2022 and 2024 were retrospectively collected. The Southend Pre-Test Probability Score for GCA (GCAPS) was calculated. Patients underwent color-Doppler ultrasound (CDUS) for the temporal and axillary arteries. The definitive diagnosis was determined by the treating rheumatologist based on clinical features, CDUS, additional imaging tests, and/or biopsy results.

**Results:**

Seventy-two consecutive outpatients with a suspected diagnosis of GCA were included. The mean age was 74.6 ± 8.8years, 63% were females. Of these individuals, 19/72 had a low GCAPS, of whom 2 were diagnosed with GCA; 40/72 had an intermediate GCAPS, of whom 14 had a positive CDUS but only 9 had GCA diagnosis; and of 13 patients with high GCAPS, 10 were confirmed as having GCA by CDUS and one patient was diagnosed by computed tomography angiography (CTA). Large-vessel involvement was documented in only 2/22 GCA patients. Among non-GCA patients, the most common diagnoses were pure polymyalgia rheumatica (PMR), migraine headache, infection, calcium-pyrophosphate deposition disease (CPPD), and temporomandibular joint (TMJ) dysfunction.

**Conclusions:**

The GCAPS is a reliable tool for risk-stratifying patients referred to the GCA-FTC in this study population. The sensitivity and specificity of CDUS was 90.9% and 88%, respectively. A relatively low prevalence of large vessel involvement was documented. Our results suggest that rheumatologists should remain vigilant regarding the differential diagnoses in patients with suspected GCA.

**Table:** 

**Key Points** • *Our study is the first study in the Middle East to utilize color-Doppler ultrasonography (CDUS) in Giant Cell Arteritis Fast Track Clinic (GCA-FTC) setting, and it was found to have very high sensitivity (90.9) and specificity (88.0%).* • *In our study population, the Southend Pre-Test Probability Score for GCA (GCAPS) was found to be a reliable tool for risk- stratifying patients referred to the GCA-FTC.* • *Based on our findings, rheumatologists should remain vigilant regarding the differential diagnoses of patients with suspected GCA, including pure PMR, migraine headache, infection, CPPD and TMJ dysfunction.*

**Supplementary Information:**

The online version contains supplementary material available at 10.1007/s10067-025-07787-0.

## Introduction

The incidence of Giant cell arteritis (GCA) varies among different geographic regions and ethnic origins. Epidemiological studies from the Middle East and non-European countries, including Israel [1, 2], Jordan [3], and Saudi Arabia [4] Tunisia [5] and northwestern Turkey [6] have collectively reported a relatively lower incidence rate of GCA compared to the global and north European rates [7, 8] and with regional variation in age and sex distribution at diagnosis. None of these studies used ultrasound in patients’ assessment, and no data was provided on the disease phenotype.

The 2023 European Alliance of Associations for Rheumatology (EULAR) recommendations advise on the use of ultrasound as first-line imaging test in all patients with suspected giant cell arteritis [9]. Ultrasound is a widely available device that allows for the rapid, relatively inexpensive, and patient-friendly evaluation of patients and provides excellent reliability, yielding pooled sensitivities and specificities of 88% (95% CI 82%–92%) and 96% (95% CI 86%–99%), respectively, as reported in a study by Bosch et al. [10]. Accordingly, Fast Track Clinics for early diagnosis of GCA (GCA-FTC) utilizing vascular ultrasound were established and were shown to have led to a substantial reduction in visual loss [11], where ultrasound was also shown to be potentially useful as a monitoring tool in patients with GCA [12]. Vascular imaging identifies different clinical subsets or phenotypes of GCA based on the involvement of temporal and/or extracranial arteries [13]. These GCA subsets differ in clinical presentation and prognosis. However, data on Middle Eastern patients referred to GCA-FTC for evaluation, and the correlation between their clinical presentation and disease subsets or phenotypes of GCA, is lacking.

In this study, we aim to present our experience from our GCA-FTC by investigating the demographic, clinical, and imaging characteristics, as well as to provide the definitive diagnoses of patients referred for evaluation at a GCA-FTC in the northwest of Israel, aiming to learn more about the disease expression and phenotype of GCA in our region, which is comprised of diverse ethnic groups.

## Methods

### Patient population

Clalit Health Services (CHS), which is the largest healthcare provider in Israel, serves approximately 4.9 million members constituting ~ 52% of Israel’s population. The CHS membership is comprised of individuals of widely diverse geographic distribution, different ethnicities, and from all socioeconomic backgrounds across Israel, with all members having equal access to the same uniform medical benefits. Within CHS, Carmel Medical Center (CMC) serves as a referral center for patients living in the Haifa and West Galilee District in northwestern Israel. This district serves about 790,000 CHS enrolees, 62% of whom are Jewish and 38% of whom are Arab.

In 2021, the Carmel Medical Center Giant Cell Arteritis Fast Track Clinic (CMC GCA-FTC) was established to serve as a site for the rapid examination of patients suspected of having GCA by an experienced rheumatologist, who is responsible for taking the necessary medical history, as well as performing the necessary clinical and color-Doppler ultrasound (CDUS) evaluations of referred patients to arrive at a reliable diagnosis of GCA. Patients with suspected GCA enrolled in this study were initially assessed as outpatients in three CHS general rheumatology clinics, numbering twelve referring rheumatologists or during in-house rheumatology consultations. Additionally, in some cases with suspected direct end-organ involvement, these patients were referred directly to the CMC GCA-FTC for evaluation by other physicians, including primary care physicians, neurologists, and ophthalmologists. Notably, the common practice in our region is to refer all patients with suspected GCA to rheumatology evaluation, so that any case suspected of vasculitis is seen by a rheumatologist. Within the CMC GCA-FTC, patients were then re-evaluated by a rheumatologist to confirm the medical history, and to perform both the clinical and vascular CDUS examinations of these patients, with patients found to have GCA or pure PMR referred back to the treating rheumatologist and to any additional subspecialist (i.e. ophthalmologist, neurologist, vascular surgeon ears-nose-throat specialist, or cardiologist) based on the patient’s organ involvement.

In this study, demographic, clinical, and laboratory data of patients referred to the CMC GCA-FTC between January 2022 and December 2024 were retrospectively collected. The Southend Pre-Test Probability Score for GCA (GCAPS) developed by Laskou et al. [14] was used to calculate the pre-test probability for GCA in all patients. Briefly, GCAPS serves as a risk assessment tool, incorporating demographic characteristics such as biological sex and age, symptom duration, clinical characteristics of symptoms (i.e. presence of headache and polymyalgic, constitutional and/or ischemic symptoms), presence of clinical signs of GCA (i.e. characteristic visual loss, temporal artery abnormality, and/or cranial nerve palsy), and C-reactive protein (CRP) level. The validity of this score was tested by Melville et al. [15] and the overall performance of GCAPS in distinguishing GCA/non-GCA was shown to be excellent [area under the receiver operating characteristic (ROC) curve, 0.976 (95% CI 0.954, 0.999)], highlighting its utility as a risk assessment and a diagnostic tool. In this scoring system, GCAPS < 10 was found to be sufficient to exclude GCA without further evaluation [14, 15].

All patients in the CMC GCA-FTC underwent CDUS evaluation of the common superficial temporal artery and its frontal and parietal branches as well as the axillary arteries bilaterally, using a modern high-quality machine (PHILIPS AFFINITY 70) equipped with a linear high frequency probe (el-Philips, 18-4MHZ), using pre-defined settings for both the cranial and extracranial arteries. A positive CDUS examination was reported in cases where a halo sign was observed, or upon documentation of an increased intima media thickness in any of the scanned segments as defined in the literature [13, 16]. Each patient with a positive CDUS was referred to CTA for further large vessel imaging in order to ascertain extent of large vessel involvement, with patients referred to additional testing for large vessel evaluation [magnetic resonance imaging (MRI), positron emission tomography (PET)-CT and/or temporal artery biopsy (TAB)] in equivocal cases. Following evaluation in the CMC GCA-FTC, patients completed additional medical evaluations as needed, according to the judgment of the referring/treating physician in cases of suspicion of alternative diagnoses. The definitive diagnosis in each referred case was determined within 1–3 months by the referring/treating rheumatologist based on clinical features, CDUS findings, any additional imaging tests, and/or TAB results, in accordance with the 2022 American College of Rheumatology (ACR)/EULAR classification criteria for giant cell arteritis [17].

### Statistical analysis

Continuous variables were summarized as mean ± standard deviation (SD), while categorical variables were presented as numbers and proportions. Comparisons of baseline characteristics between patients diagnosed with GCA and patients evaluated in our CMC GCA-FTC without diagnosis of GCA was done using chi-square test for categorical variables and using Student’s *t*-test for continuous variables, as appropriate. In all analyses, *P* ≤ 0.05 for the 2-tailed tests was considered statistically significant. Sensitivity and specificity were calculated to evaluate the performance of ultrasound findings for the diagnosis of GCA.

The overall performance of GCAPS in distinguishing GCA from non-GCA was evaluated in our patient population using ROC curve analysis followed by Youden index. A GCAPS score $\ge 9$ (intermediate and high pretest probability) was utilized for this calculation. Multilevel likelihood ratios (+ LRs, − LRs) were calculated for each GCAPS category (low/intermediate/high).

All statistical analyses were performed using IBM SPSS statistics vs. 28. (IBM, New York, NY, USA).

### Ethical considerations

This study was performed in accordance with the principles of the Declaration of Helsinki and was approved by the research ethics committees (institutional review board) of Carmel Medical Center (CMC-0135–24).

## Results

**Fig. 1:**
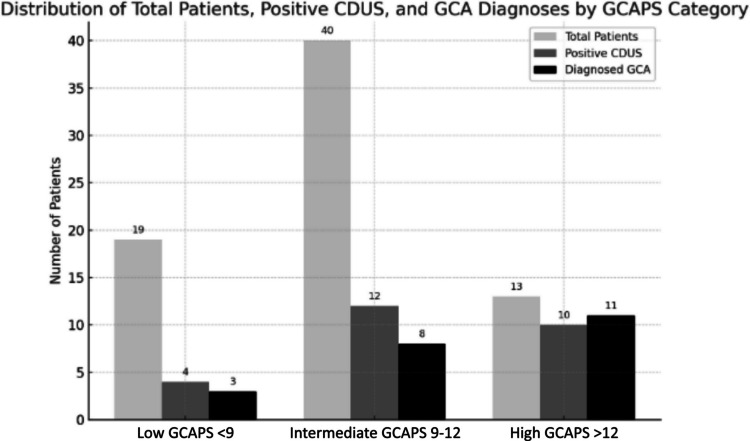
Distribution of study patients stratified by GCAPS category, CDUS results and GCA diagnosis

**Fig. 2:**
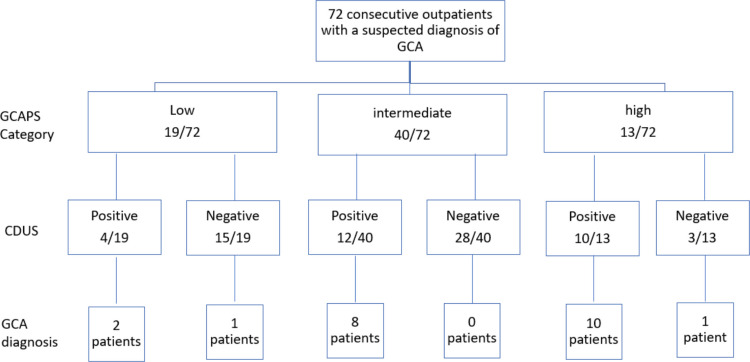
A flow diagram presenting the diagnostic pathway of study population stratified by GCAPS category

Seventy-two consecutive patients with a suspected diagnosis of GCA were referred to the CMC GCA-FTC from January 2022 until December 2024 to complete CDUS examination and constituted the study population. The mean age was 74.6 ± 8.8 years, with 63% female patients and 83.3% identified as Jewish. Of patients, 22/72 were diagnosed with GCA by the treating rheumatologist based on clinical, laboratory and imaging findings. Among the patients, 19/72 had a low GCAPS, of whom 2 had a positive CDUS and 3 were diagnosed with GCA; 40/72 had an intermediate GCAPS, of whom 14 had a positive CDUS and of whom only 8 were diagnosed with GCA. Patients with high GCAPS (13/72) included 11 confirmed GCA cases of which ten were confirmed by positive CDUS and one case diagnosed by CTA, yielding an overall sensitivity of 90.9%, 95%CI (72.2–97.5), and a specificity of 88.0%, 95%CI (76.2–94.4) for CDUS for the detection of GCA. Of 8 patients with palpable temporal artery abnormalities, 7/8 also had corresponding CDUS positivity characterized by a positive halo sign along with documentation of an increased intima media thickness. The patients’ distribution is detailed in Figs. 1 and 2 and Table 1. Among the 72 patients, TAB was performed in 9 cases, and only one was positive. Notably, this patient also had a concordant positive CDUS result. None of the 8/9 patients with a negative biopsy had a positive CDUS and none were diagnosed with GCA.

**Table 1:** Characteristics of patients referred to CMC GCA-FTC with GCA diagnosis

	Patients with GCA [*N *= 22, (%)]	Patients without GCA [*N *= 50, (%)]	(***p***-value)
Mean age	76.8 ± 7.7	73.6 ± 9.2	0.160
Sex (Females)	12/22 (54.5%)	34 (68.0)	0.274
Ethnicity (Jewish)	20 (91%)	44 (88.0)	> 0.99
(Arab)	2 (9%)	6 (12%)	
Duration of symptoms			
< *6 weeks*	19/22 (86.4%)	23 (46.0)	0.008
* 6–12 weeks*	2/22 (9.1%)	4 (8.0)	
* 12–24 weeks*	1/22 (4.5%)	5 (10)	
> *24 weeks*	0%	18 (36.0)	
C-*reactive protein (CRP)*			
* 0–5 mg/L*	2/22 (9.1%)	12/50 (24.0)	0.041
* 6–10 mg/L*	1/22 (4.5%)	9/50 (18.0)	
* 11–25 mg/L*	5/22 (22.7%)	14/50 (28.0)	
> *25 mg/L*	14/22 (63.6%)	15/50 (30.0)	
Mean ESR (mm/h)	*N* = 20 69 ± 29 70 (45; 90)	*N* = 49 50.4 ± 37.5 35 (23; 89)	0.015
Headache	19/22 (86.4%)	34 (68.0)	0.103
Polymyalgia Rheumatica (PMR)	8/22 (36.4%)	23 (46.9)	0.406
Constitutional symptoms	7/22 (31.8%)	7 (14.3)	0.111
Ischemic symptoms	6/22 (27.2%)	1 (2.0)	0.003
Visual symptoms (AION, CRAO, Field loss, RAPD)	4/22 (18.2%)	4 (8.0)	0.237
Palpable temporal artery abnormality	8/22 (36.4%)	2 (4.0)	< 0.001
Extracranial artery abnormality	0%	2 (4.0)	> 0.99
Cranial nerve palsy	1/22 (4.5%)	2 (4.0)	> 0.99
GCAPS category			
Low probability GCAPS score	3 (9%)	27 (54.0)	< 0.001
Intermediate probability GCAPS score	8 (41%)	21 (42.0)	
High probability GCAPS score	11 (50%)	2 (4.0)	

*AION* anterior ischemic optic neuropathy, *CRAO* central retinal artery occlusion, *ESR* erythrocyte sedimentation rate, *RAPD* relative afferent pupillary defect

One patient with low GCAPS score and negative CDUS was diagnosed with GCA based on positive CT imaging of aortitis.

The overall performance of GCAPS in distinguishing GCA from non-GCA was evaluated using ROC curve analysis. A GCAPS score $\ge$ 9 (intermediate and high pretest probability) yielded an area under the ROC curve (AUC) of 0.816 (95% CI: 0.697–0.936), underscoring the strong discriminative ability and clinical utility of this tool for risk assessment and diagnostic support (Supplementary Fig. 1). Moreover, the likelihood ratios were calculated for each GCAPS category and showed a clear, direct correlation between higher GCAPS scores and the probability of GCA. Specifically, for patients with low and intermediate GCAPS scores, the positive likelihood ratio (+ LR) was 0.25 and 0.86, respectively, in contrast to patients with high GCAPS scores who demonstrated a markedly increased + LR of 12.5 (Supplementary Table 1). These findings demonstrate a strong positive correlation between GCAPS category and the likelihood of GCA, supporting its clinical usefulness as a risk stratification tool.

Table 1 presents disease characteristics and subsets of the 22 patients diagnosed with GCA. Most of the patients (90.1%), had an intermediate to high pre-test probability GCAPS. The mean age of patients diagnosed with GCA was 76.8 ± 7.7 years, of whom only 54% were females. Most of the patients (86.4%) had symptom duration of less than 6 weeks, and the majority (63.6%) had a CRP level above 25 mg/L (normal range 0–5 mg/L) with a mean ESR of 69 ± 29 mm/h. The majority of patients presented with headache (86.4%) and only 36.4% had PMR symptoms, whereas 31.8% had constitutional symptoms. Ischemic symptoms (4/6 with jaw claudication and 3/6 with visual symptoms) were present in 27.2% of patients with GCA and visual symptoms in 18.2% of patients with GCA. Patients without GCA (N = 50), differed in terms of disease duration, C-reactive protein and ESR levels as well as in distributions in the GCAPS scoring categories compared to patients with GCA, as shown in Table 1.

Large vessel involvement was documented in only 2/22 (9.1%) of patients with GCA. Among non-GCA patients (*N* = 50), the most common diagnoses within the period of follow-up were pure PMR (18/50), migraine headache (10/50), infection (4/50) (notably sinusitis, pneumonia, viral infection and urinary tract infection), CPPD presenting as Crowned Dens Syndrome (3/50) and patients with TMJ dysfunction (3/50). No diagnosis was made in 5/50 (10%) patients, and these individuals were all asymptomatic at follow-up clinic visit. Other diagnoses included acute thyroiditis, sarcoidosis and post-traumatic injury (post craniotomy). Of the 6 patients with a false positive CDUS test, the final diagnosis remained unknown in 2 patients, as all had complete resolution of their symptoms with a short glucocorticosteroid taper and no additional workup was performed regarding their initial presentation. Of the other 4 patients with false positive CDUS test, two patients were diagnosed with PMR, a single patient was diagnosed and treated for a migraine headache with prompt resolution of migraine symptoms, and one patient was diagnosed with antiphospholipid antibody syndrome.

## Discussion

In this study, we present the patient population who was referred to the CMC GCA-FTC between January 2022 and December 2024 for CDUS evaluation due to suspected GCA. The GCAPS pre-test probability score assigned to the study population was moderate to high in most patients, and this score correlated well with the final diagnosis of GCA (91% of cases). Therefore, our data shows that GCAPS serves as a useful tool for risk-stratifying patients suspected of having GCA in the GCA-FTC setting. Similarly, the utility of the GCAPS score combined with ultrasonography in excluding GCA from GCA mimics has recently been shown in a prospective multi-center European study on GCA-FTC [18].

In our study, the sensitivity and specificity of the CDUS exam were also found to be high and comparable to what is reported in the literature [19], thus supporting the diagnostic accuracy of this tool. High diagnostic accuracy can be obtained using CDUS especially when an experienced sonographer uses a modern ultrasound probe thanks to the high reliability of the elementary lesions [19] that are sought after in the CDUS exam, and when using the EULAR-OMERACT Giant cell arteritis Ultrasonography Score (OGUS) [20].

To our knowledge, this is the first study to shed a light on the phenotypic subset of GCA patients from the northwest of Israel, with notably only 2/22 patients having evidence of extracranial large vessel involvement, in contrast with reported data from the Berlin-Buch FTC, in which out of 367 patients with GCA, 48% had cranial involvement, 21% had extracranial involvement, and 32% had mixed large vessel and cranial GCA, [19] suggesting that overall, approximately half of GCA patients have large vessel involvement. Along these lines, data from the Diagnostic and Classification Criteria in Vasculitis (DCVAS) international cohort [13] also report a higher percentage (23%) of GCA patients having large vessel involvement, either as pure extracranial large vessel involvement, or as part of mixed cranial and extracranial GCA. A possible explanation for this variation other than ethnic/regional variation might be referral bias given that patients were being sent to our GCA-FTC primarily for CDUS evaluation, which would help diagnose patients primarily with cranial artery abnormalities. It is less likely that this variation would stem from imaging threshold or equipment differences as the equipment and visualization protocol that we used were standardized, as noted in the methods section above.

An additional finding of interest in our cohort is the lack of female sex predominance for disease (54% of the patients were females), in contrast with reports from Western countries, in which the incidence of GCA is approximately 3:1 in women relative to men [8]. The low percentage of Arab patients found to have GCA in our cohort (9%) is also of interest, especially considering that this patient population constitutes approximately 38% of CHS enrolees, and is in keeping with data published in the literature on the Arab world [3–5]. Given the small number of patients diagnosed with GCA in our cohort, continued evaluation of additional patient referrals would help elucidate whether these demographic differences are real.

Based on our findings, rheumatologists should remain vigilant regarding the differential diagnoses of patients with suspected GCA, including migraine headache, infection, TMJ dysfunction and Crowned Dens Syndrome. We encountered these challenging differential diagnoses in our GCA-FTC. Moreover, it is of interest whether the 18/50 patients who were confirmed with pure PMR in our study would develop subclinical GCA on later follow-up, as current literature reports that about 23% of patients with PMR actually have concurrent subclinical GCA as noted on initial CDUS evaluation [21] or by other diagnostic tests [22]. In addition, rheumatologists should be aware of other mimickers of disease [23] as well as possess awareness of the fact that not every patient presenting with a positive halo sign or increased intima media thickness actually has GCA, as we found in our study population.

Despite our interesting findings, our study has some limitations. Given the relatively small number of patients who were enrolled in the study, and the small number of confirmed GCA cases, our study findings should be interpreted with caution but advocate for further research to generalize our study findings. Unfortunately, as not all enrolees in CHS were assessed by our particular GCA-FTC, no data could be derived in our study on the prevalence of GCA in the entire northwestern region of Israel, and we also lacked data dividing our Jewish population into those of Ashkenazi vs Sephardic ancestry. Therefore, more research is required to investigate whether GCA is genuinely relatively equally prevalent in men and women in our geographical region, and whether its prevalence is indeed low in our Arab population and on its prevalence in various subgroups of the Jewish population. Future prospective, multi-center studies with larger sample sizes and long-term follow-up are needed to confirm the disease subsets and demographic findings from our study. This long-term follow-up would be particularly important in cases where pure PMR is initially diagnosed and GCA is diagnosed later, when glucocorticosteroid taper is unsuccessful and further imaging is conducted to evaluate again for GCA, and in our study was variable depending on when the patient was initially evaluated for GCA during the study timeframe of January 2022–December 2024.

To conclude, this study, which is the first study in the Middle East to utilize CDUS in GCA-FTC setting, contributes valuable insights into regional variations in GCA presentation, reinforces the importance of ultrasound as a diagnostic tool and the need for a careful clinical-imaging correlation in suspected GCA patients, and suggests that GCAPS can effectively risk-stratify suspected GCA cases. Additional research on GCA in diverse populations is important as it could refine future diagnostic approaches and provide a better understanding of the disease in diverse populations.

## Supplementary Information

Below is the link to the electronic supplementary material.

ESM 1 Supplementary Material 1 (DOCX 40.9 KB)

## Data Availability

The data used and/or analyzed during the present study are available from the corresponding author on reasonable request.
