# Supplementary material for: Patient characteristics, disease manifestations and diagnostic findings of consecutive patients suspected of Giant Cell Arteritis (GCA) — retrospective experience from a fast-track clinic in Israel
Source: Clin Rheumatol. 2025 Nov 27;45(2):1181–7. doi: 10.1007/s10067-025-07787-0 (PMC12858618; doi:10.1007/s10067-025-07787-0)
Supplement: Supplementary file 1 — Supplementary Material 1 (DOCX 40.9 KB) [file 10067_2025_7787_MOESM1_ESM.docx]

**Supplementary material**


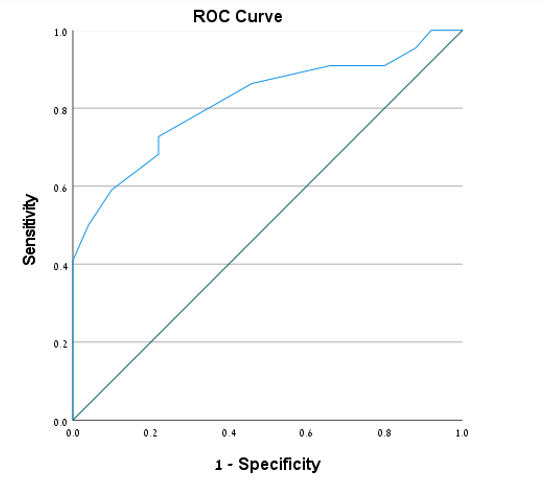


**Supplementary Figure 1**. The overall performance of GCAPS in distinguishing GCA from non-GCA was evaluated using receiver operating characteristic (ROC) curve analysis. A GCAPS score $\geq9$ (intermediate and high pretest probability) yielded an area under the ROC curve (AUC) of 0.816 (95% CI: 0.697–0.936), underscoring the strong discriminative ability and clinical utility of this tool for risk assessment and diagnostic support.

**Supplementary Table 1:** The likelihood ratios for each GCAPS category

| LR | GCA negative | GCA positive |  |
| --- | --- | --- | --- |
| 0.25 | 27/50=0.54 | 3/22=0.136 | Low <9 |
| 0.86 | 21/50=0.42 | 8/22=0.36 | Intermediate 9-12 |
| 12.5 | 2/50=0.04 | 11/22=0.50 | High >12 |
